# Detection of hypoxia markers in the cerebellum after a traumatic frontal cortex injury: a human postmortem gene expression analysis

**DOI:** 10.1007/s00414-014-1129-3

**Published:** 2014-11-29

**Authors:** K. Schober, B. Ondruschka, J. Dreßler, M. Abend

**Affiliations:** 1 German Air Force Center for Aerospace Medicine, Postfach 1264/KFL, 82242 Fürstenfeldbruck, Germany; 2 Institute of Legal Medicine, Medical Faculty, University of Leipzig, Johannisallee 28, 04103 Leipzig, Germany; 3 Bundeswehr Institute of Radiobiology, Neuherbergstraße 11, 80937 Munich, Germany

**Keywords:** Traumatic brain injury, Human, Post mortem, Gene expression, Biomarker, mRNA, miRNA, IL-6, FOSB, HSPA12B, HSD11B1, miR-138, miR-744, miR-195, miR-324-5p

## Abstract

**Purpose:**

The response to traumatic brain injury (TBI) is complex and induces various biological pathways in all brain regions that contribute to bad outcomes. The cerebellar hypoxia after a frontal cortex injury may potentiate the pathophysiological impacts of TBI. Therefore, a gene expression analysis was conducted to determine the influence of hypoxia on TBIs.

**Material and methods:**

Total RNA, including microRNAs, was isolated from the cerebellum of individuals who had died from severe frontal cortex injuries or due to natural causes of death (reference group).

**Results:**

From a total of 19,596 genes, an average of 59.56 % messenger RNAs (mRNAs) appeared expressed with 42 of them showing significant >2-fold differences of upregulated (*n* = 18) and downregulated (*n* = 24) genes. The validity of 14 candidate genes (with low *p* values and high fold differences or based on cited literature) was confirmed using qRT-PCR (Spearman correlation *r* ^2^ = 0.93). Only four genes appeared to be either upregulated (FOSB and IL6) or downregulated (HSD11B1 and HSPA12B). From a total of 667 microRNAs, altogether, 248 microRNAs appeared expressed with 13 of them showing significant differences in the mean gene expression. The combination of two mRNAs (HSPA12B/FOSB or IL6/HSD11B1) or two microRNAs (either miR-138/miR-744 or miR-195/miR-324-5p) completely discriminated both groups, a finding unaltered by potential confounders such as age at biosampling, survival time, and the postmortem interval.

**Conclusions:**

Cerebellar hypoxia markers are important to understand the pathophysiology of TBIs and could be used for therapeutic strategies or forensic purposes, e.g., to assess the severity of a brain injury.

**Electronic supplementary material:**

The online version of this article (doi:10.1007/s00414-014-1129-3) contains supplementary material, which is available to authorized users.

## Introduction

Traumatic brain injury (TBI) is among the most frequent causes of death after traffic accidents or assaults [1, 2].

The primary impact can promote secondary damages with the extent depending upon injury severity. Secondary events can lead to cerebral hypoxia that induces the anaerobic glycolysis pathway resulting in energy depletion. This promotes the neurotoxic cascade like the energy-dependent ion pumps failure, acidosis, membrane depolarization, the influx of calcium and sodium, the release of glutamate, and the activation of apoptosis and inflammation [3–8]. The secondary events compromise a not well-understood multi-cellular system involving hundreds of interacting components.

Gene expression profiling has the capability to identify pathways and novel biomarkers which are associated with cerebral hypoxia following TBI.

It is known that small RNAs, named microRNA (miRNA), are involved in the regulation of gene expression after TBI [9–13]. So far, about 940 different miRNA species have been detected in humans [14, 15]. Prominent features of miRNAs are the tissue specificity and disease-specific patterns [16, 17]. However, studies on human postmortem whole-genome transcriptional changes (mRNAs) and post-transcriptional miRNA gene expression changes that are associated with cerebral hypoxia, especially in the cerebellum, are missing.

The cerebellum was chosen because it reacts vulnerably to hypoxia [18, 19] and could contribute to the TBI pathophysiology.

Neurological deficits in the cortex following TBI are correlated with a loss of motor control and coordination; these properties are associated with cerebellar functions [20]. It could be shown that a forebrain injury resulted in a loss of Purkinje cells in the cerebellum [21]. It was supposed that the excitotoxic release of glutamate is involved in the Purkinje cell death following a forebrain injury [22]. However, the causes of indirect cerebellar damages in areas that are distant from the injured site are not completely understood.

The main question was if it would be possible to find biomarkers for the identification of hypoxia in the cerebellum after a frontal a cortex injury (violent death) in comparison to the reference group (natural death) based on gene expression changes considering potential confounders such as age at biosampling, survival time (used as a surrogate for the severity of the cranial trauma), and the postmortem interval time (time interval between the person’s death and the tissue sampling).

## Material and methods

### Autopsy cases

We used small brain sections from deceased humans that were taken during the medicolegal investigation of the deceased. The Leipzig public prosecutor’s office allowed us to analyze these samples to determine the cause of death according to the guidelines from the central ethics commission of the German Medical Association. Following the prerequisites of the ethics commission (e.g., use of anonymized samples, no ethically questionable study, and no evidences for rejection from the deceased), our study became approved in 2012 by the local ethics commission of the Medical Faculty of the Leipzig University (AZ: 117-12-23012012).

We included male Caucasians aged between 19 and 80 years (median TBI, 50 years; reference group, 57 years). The causes of injury in the TBI group (*n* = 8) were one fall, two household accidents, and five motor vehicle accidents. The TBI group comprised individuals with a frontal cortical contusion zone and cranial traumata as the cause of death. The survival time in the TBI group varied between <2 and 18 h (mean, 4 h).

In the reference group (*n* = 7), there were no brain contusions and death had occurred within less than 2 h because of other reasons, namely myocardial infarction, ischemic heart disease, pulmonary embolism, ruptured aneurysm, or pneumonia.

The postmortem interval in both groups varied between 29 and 132 h (median, 58 h).

We excluded individuals due to toxicological reasons (drug and alcohol consumption), histological damages to the brain (cerebellum contusion in the cranial trauma group and medical history of cranial trauma in the reference group), or known genetic and neurological diseases. None of the cases showed any signs of neoplasm (for details see Table 1).

**Table 1:** Details of trauma and control cases

	Age	Cause of injury	Cause of death	Survival time (h)	PMI (h)
Trauma cases	50	Fall	Brain injury	<2	48
	58	Household accident	Brain injury	<2	87
	19	Motor vehicle accident	Brain injury + multiple injuries	<2	60
	19	Motor vehicle accident	Brain injury	<2	29
	49	Motor vehicle accident	Brain injury + multiple injuries	<2	64
	49	Motor vehicle accident	Brain injury	3	50
	74	Motor vehicle accident	Brain injury	8	113
	80	Household accident	Brain injury	18	132
Control cases	57	Collapsed suddenly	Myocardial infarction	<2	95
	47	Collapsed suddenly	Ruptured aneurysm	<2	47
	44	Collapsed suddenly	Ischemic heart disease	<2	52
	51	Collapsed suddenly	Myocardial infarction	<2	63
	71	Pulmonary embolism	Ischemic heart disease	<2	67
	68	Collapsed suddenly	Myocardial infarction	<2	43
	75	Collapsed suddenly	Pneumonia	<2	43

### Tissue samples and histological examination

Samples of prepared semisterile cerebellar tissue were taken after death, cryopreserved in nitric oxide, and stored at −80 °C before use. All tissue samples were examined by an experienced pathologist for histological classification. For that reason, the tissue sections were stained using hematoxylin and eosin to evaluate the morphology and possible traumatic changes. None of the TBI cases showed any traumatic damages such as bleedings or tissue lacerations, cerebellar edemata (loosening of Bergmann-Glia, spongiotic swelling, and pericellular edema), or necrotic areas. Furthermore, no relevant histomorphological differences between the TBI and control cases were found.

### RNA isolation

Frozen biopsy pieces were incubated into 2 ml ceramic bead tubes (1.4 mm; Süd-Laborbedarf GmbH, Gauting, Germany) filled with 700 μl QIAzol® Lysis Reagent (Qiagen, Hilden, Germany), carefully thawed, and homogenized (PowerLyzer^TM^24, Süd-Laborbedarf GmbH, Gauting, Germany). Total RNA, including small RNAs, was isolated (miRNeasy Mini Kit (Qiagen, Hilden, Germany) and the remaining DNA was digested. RNA was stored at −80 °C until its use. Quality and quantity of isolated total RNA were measured spectrophotometrically (NanoDrop, PeqLab Biotechnology, Erlangen, Germany) while the RNA integrity number (RIN) was assessed by the 2100 Agilent Bioanalyzer (Life Science Group, Penzberg, Germany). Aliquots of samples with suspicious RIN 6.0–7.0 (*n* = 6) were incubated for 1 h at 37 °C to test for the presence of RNases. The RNA integrity (28/18S ribosomal RNA (rRNA) bands) was verified by agarose gel electrophoresis. All analyzed samples showed unaltered 28/18S bands without any signs of degradation (smear) and were forwarded for both whole-genome microarray analysis and qRT-PCR. All samples passed the multiple quality controls inherent to both methods.

### Whole-genome microarray and data analysis

Genome-wide gene expression profiling was carried out using the Agilent oligo microarray GE 8x60K v2 (Agilent Technologies, Waldbronn, Germany) combined with a one color-based hybridization protocol. Signals on the microarray were detected using the Agilent DNA Microarray Scanner. GeneSpring GX12.5 software was employed to quantile normalize the raw data. We analyzed gene expression as an outcome and compared gene expression between both groups using the non-parametric Welch’s approximate *t* test. Genes had to be expressed in at least 50 % of the samples per group. Only significant and ≥2-fold differences in gene expression among groups were considered for comparisons, and unadjusted *p* values and *p* values adjusted for multiple comparisons (false discovery rate) were calculated. All gene candidates underwent gene set enrichment analyses using PANTHER pathway software (http://www.pantherdb.org/) that groups genes with similar biological functions based on their annotation. For methodological validation purposes, we selected 14 genes showing low *p* values and high fold changes (*n* = 10) or TBI markers based on cited literature (*n* = 4). These genes were examined on the same samples but using qRT-PCR which provides quantitative data. These data were finally compared with semi-quantitative whole-genome microarray data for methodological comparison purposes and analyzed employing the logistic regression analysis.

### Quantitative RT-PCR experiments

We employed TaqMan chemistry (Hs-code in parenthesis) for the detection of our ten mRNA gene candidates which are ARPC5 (hs00271722_m1), BCAT1 (Hs00398962_m1), FOSB (Hs00171851_m1), GADD45B (Hs00169587_m1), GRIA3 (Hs01557466_1), HSD11B1 (Hs01547870_m1), HSPA12B (Hs00369554_m1), IL6 (Hs00985639_m1), PRPH (Hs00196608_m1), and RGS6 (Hs00188763_m1).

Based on cited work [23], we added another four markers to our analysis which are CASP3 (Hs00234387_m1), GFAP (Hs00909233_m1), NTRK2 (Hs00178811_m1), and S100B (Hs00902901_m1). The RNA samples were processed by converting 5 μg of total RNA into cDNA (High Capacity Kit) and running a PCR protocol according manufacturer’s instructions (Life Technologies GmbH, Darmstadt, Germany) For all transcripts, we tested 18S rRNA (Hs03928985_g1), GAPDH (Hs99999905_m1), and HBMS (Hs00609297_m1) for normalization purposes.

Altogether, 667 miRNA could be analyzed employing two 384-well LDAs (LDA type A&B). Per LDA type, a 2-μg RNA aliquot of each RNA sample was analyzed according manufacture instructions (Life Technologies GmbH, Darmstadt, Germany).

All technical procedures for qRT-PCR were performed in accordance with standard operating procedures implemented in our laboratory in 2008 when the Bundeswehr Institute of Radiobiology became accredited according to DIN EN ISO 9001/2008. Ahead of our experiment, we had established the upper limit of the linear-dynamic range of our qRT-PCR using replicate measurements on one of our miRNA samples. The upper limit occurred at Ct ≤26 (cycle threshold, Ct). Ct values were normalized relative to the median gene expression of the examined miRNAs. This approach to normalization was more robust compared to the use of the internal control (data not shown).

### Statistical analysis of quantitative RT-PCR mRNA and miRNA data

Normalized Ct values of our miRNA and mRNA candidate genes must be expressed in at least 50 % of the samples in one group. We compared the TBI group and the control group by examining statistical differences using either the *t* test or the Mann–Whitney rank sum test where appropriate. Significant candidate genes were finally examined using the logistic regression analysis. By means of the logistic regression analysis, a prediction model and a weighting factor (maximum likelihood estimate) were calculated for each parameter so that the contrast between both groups became amplified. Predictions of this model were compared with the known group members, and concordant pairs of predicted probabilities and observed responses were computed using the Hosmer-Lemeshow goodness-of-fit test. Concordances of 100 % indicated a complete agreement of the model with the known groups and, therefore, a complete discrimination of TBI from the reference group.

These analyses were run separately for each single miRNA and our selected 14 mRNA candidate genes (univariate analysis). Finally, we conducted a multivariate analysis to examine whether a combination of variables might improve our results, employing forward and backward selection procedures. This was done for complete data sets only and, separately, for the miRNA and mRNA genes to be able to better examine potential candidates originating from the transcriptional or post-transcriptional level.

In a final step, we separately adjusted our logistic regression models for each of three potential confounders (age at biosampling, survival time, and the postmortem interval). At first, we examined the impact of the confounder on the discrimination of our groups (cranial trauma group versus controls). In a second step, we adjusted confounders (continuous scale) to models comprising significant associations of the groups with our candidate genes (univariate and multivariate). None of the confounders contributed significantly to these models and they were hence excluded from further analysis (data not shown).

## Results

### RNA isolation

On average, we isolated 33.6 μg of total RNA (SD ±14.11; range, 8.65–62.22) from 10 to 20 mg of our biopsy samples. The average RIN was 7.4 (SD ±0.92; range, 6.0–9.1).

### Whole-genome microarray and PANTHER classification

From 19,596 gene mRNAs (42,545 transcripts) spotted on the whole-genome microarray, on average, 59.56 % (SD ±2.32; range, 55.0–63.5 %) was distinguishable from the background (expressed). The number of significant and differentially expressed genes (with an uncorrected *p* value of <0.05 and a 2-fold gene expression difference) was 42 with about half of them being either upregulated (18) or downregulated (24). None of these genes could sustain multiple comparisons. Due to the low number of differentially expressed genes (at least 100 genes are needed), we could not run a PANTHER enrichment analysis of biological processes such as molecular functions, cellular components, protein classes, and pathways.

### Validation of whole-genome microarray results using qRT-PCR

**Fig. 1:**
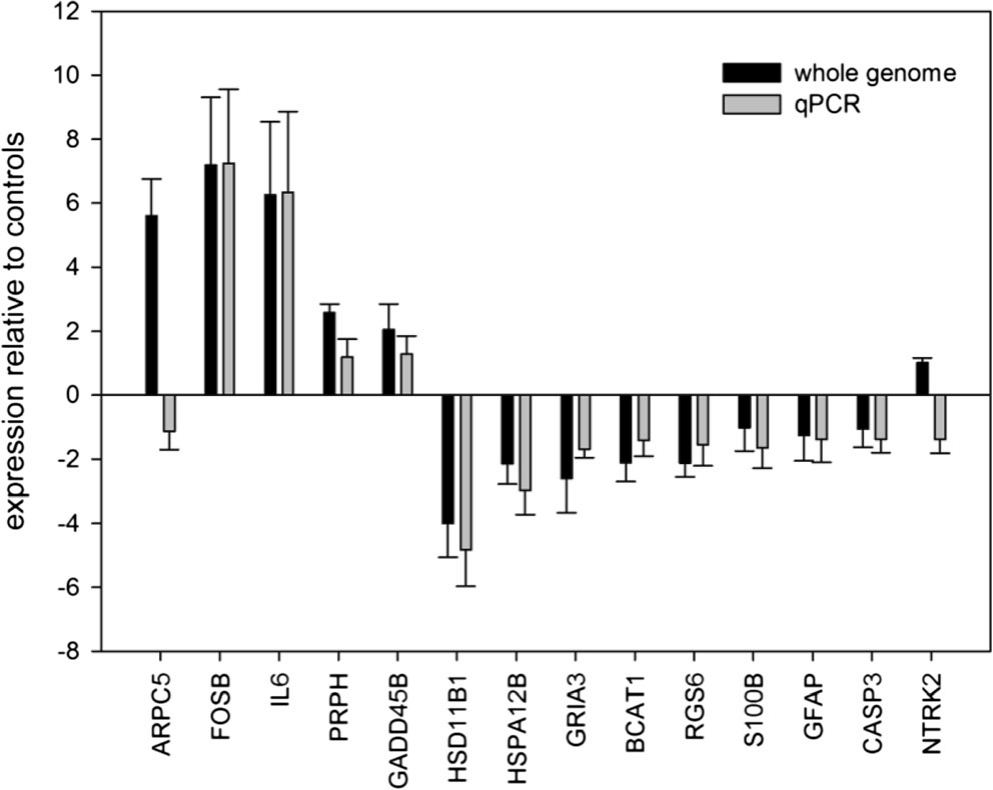
Expression of 14 differentially expressed genes (ARPC5, BCAT, CASP3, FOSB, GFAP, GADD45B, GRIA3, HSD11B1, HSPA12B, IL6, NTRK2, PRPH, RGS6, and S100B) in the cerebellum following a frontal cortex injury. *bar* refer to the mean values and *error bar* indicate the standard deviation of gene expression. Data are represented as fold change in gene expression relative to the controls

The genome microarray results were validated with qRT-PCR using 14 selected genes (Fig. 1). A linear regression was calculated over all genes except for one outlier (ARPC5). Both methods were closely correlated (*r* ^2^ = 0.93).

For qRT-PCR, we tested 18S rRNA, GAPDH, and HBMS for normalization purposes. All three markers were constantly expressed in TBI and control samples, but the 18S rRNA proved to be the most reliable among them. For instance, the main Ct values (±SD) referring to all brain samples were 20.38 ± 0.72 for 18S rRNA, 28.61 ± 0.56 for HBMS, and 21.13 ± 0.73 for GAPDH.

### Quantitative mRNA and miRNA data

The 14 mRNAs had a relative low SD (0.26 to 2.53). The sensitivity, specificity, positive and negative predictive values (PPV/NPV) as well as concordance ranged from 42.9 to 100 % (sensitivity/specificity), from 60.0 to 100 % (PPV/NPV), and from 60.7 to 96.4 % (concordance), respectively (Online Resource 1, supplemental table 1).

Ten of our genes showed control values and four genes appeared either upregulated (FOSB and IL6) or downregulated (HSD11B1 and HSPA12B). These four genes remained significantly (*p* < 0.1) associated with the two groups in the logistic regression analysis.

The combinations of two mRNAs, namely HSPA12B/FOSB and IL6/HSD11B1, completely discriminated TBI from the reference group.

From 667 miRNAs assayed, the qRT-PCR data were available for 248 miRNAs being expressed. Among them, 13 miRNAs showed a significant difference in mean gene expression per group and high consistency of data expressed by a low SD (0.10 to 0.53). We calculated the sensitivity, specificity, PPV, NPV, and concordance.

The values ranged from 57.1 to 100 % (sensitivity/specificity), from 66.7 to 100 % (PPV), from 72.7 to 100 % (NPV), and from 80.4 to 90.6 % (concordance), respectively (Online Resource 1, supplemental table 1).

The same genes were examined separately using the logistic regression analysis. *p* values became significant (*p* < 0.01). We then combined significant miRNAs to better discriminate both groups. Combinations of two miRNAs, namely miR-138/miR-744 and miR-195/miR-324-5p, completely discriminated both groups. Since we restricted this analysis to complete data sets, we loosed four genes with missing gene expression data either in the TBI group (miR-370), the reference group (miR-16), or both groups (miR-504, miR-320B).

## Discussion

The present study is the first of its kind in assessing hypoxia in the cerebellum following a frontal cortex injury in postmortem human brain tissue via a gene expression analysis.

The reversibility of secondary damages like hypoxia offers a chance for therapy to reduce cellular damage and improve a functional recovery. A prerequisite for the development of therapy strategies is the identification of the gene expression network. The study was focused on the cerebellum because of its vulnerability to hypoxia [18, 19]. Some evidence suggests that a cerebellum dysfunction after a forebrain injury contributes to TBI neuropathology [20, 21]. Park and colleagues showed a loss of Purkinje cells in the cerebellum following forebrain injuries [21]; possibly, it is the excitotoxic release of glutamate that promotes the Purkinje cell death [22].

Most human TBI studies focused their attention on protein changes in close proximity to the injury site, e.g., in the cortex, the ipsilateral cortex, the pericontusional zone, the hippocampus, or the traumatized cerebellum [23–29]. These studies detected an elevation of apoptosis [24–26, 29], stress response [28], inflammation [27], and cell cycle control [28] in the TBI group compared with the controls.

However, only few studies investigated transcriptional changes (mRNAs) of the human brain following TBI [23, 27, 30]. Frugier and colleagues found that IL-6, TNF-α, GM-CSF, and IFN-γ protein concentrations are already increased a few minutes following a TBI, indicating the important role in the early phase of an inflammatory cascade. The rise of the protein level of these pro-inflammatory cytokines correlates with increased mRNA levels [27].

This is in concordance with our results where a 6-fold IL-6 mRNA upregulation was detected in the cerebellum. Interestingly, Arand and colleagues found that IL-6 plasma levels were 8-fold higher in cases of death than those of surviving patients [31]. Furthermore, the severity of trauma correlated with the elevation of IL-6 expression [32].

Staffa and colleagues investigated apoptosis (CASP3) as well as cell cycle control (S100B), neuronal cell growth (NTRK2), and intermediate filament (GFAP) mRNA markers in traumatized cerebellum [23]. We could not detect any significant changes of these four markers in the TBI group in comparison to our reference group. Possibly, these four markers indicated a direct trauma.

Frugier and colleagues as well as Staffa and colleagues used predefined markers (top–down study) [23, 27]. In contrast, we and Michael and colleagues [30] did a whole-genome analysis (bottom–up study) of human brain tissues. Michael and colleagues found that transcription factors (c-FOS, JunB, and Zif268) and the heat shock protein Hsp70 were upregulated in the pericontusional zone of TBI patients [30].

Dave and colleagues also detected a c-fos mRNA upregulation in injured cerebellum. Interestingly, hypoxia potentiated the c-fos mRNA in the cerebellum [18]. It must be mentioned that hypoxia alone had no effect on c-fos mRNA levels, but the highest increase of c-fos was in the TBI group followed by hypoxia. This marker can be used to discriminate between hypoxia alone and hypoxia followed by a TBI. Different studies have shown that pathophysiological changes worsened when a TBI was accompanied by hypoxia [33–36].

In our study, the transcription regulator (FOSB), a member of the FOS family like c-fos, was significantly upregulated in the cerebellum despite its distance from the injured site. Furthermore, FOSB showed a direct miRNA relationship (downregulated miR-370 and upregulated FOSB; see Online Resource 1, supplemental table 2). It is known that the FosB/JunD AP-1 transcription factor complex is involved in glutamate-mediated excitotoxicity [37]. This could promote the loss of Purkinje cells in the cerebellum [22]. The FOSB marker could probably be used to detect a TBI-induced hypoxia just like the c-fos marker.

Furthermore, the increase of pro-inflammatory cytokines like IL-6 could also contribute to cell death in the cerebellum. We suppose that, on the one hand, cell death is promoted through the release of glutamate and cytokines like IL-6, but on the other hand, cell survival is promoted by preventing apoptosis through significant downregulation of HSPA12B (heat shock 70kD protein 12B; see Fig. 1). It could be shown that a downregulation of HSPA12B inhibited the expression of active caspase-3 [38]. In our study, the caspase 3 was slightly downregulated but showed a direct miRNA relationship (upregulated let-7a and downregulated caspase 3; see Online Resource 1, supplemental table 2).

Our results also showed a significant downregulation of the steroid biosynthesis marker (HSD11B1) in the TBI group in comparison to the reference group. This could contribute to an improvement of cognitive functions [39].

We assume that, despite the promotion of cell survival mechanisms, the detrimental effects are predominant in the cerebellum.

So far, only one study has analyzed post-transcriptional changes (miRNA species) in blood plasma samples from patients surviving TBI (the age varied between 14 and 65 years) [12]. In this study, miR-16 and miR-92a were used as specific markers to distinguish TBI from a polytrauma as well as a mild brain trauma from a severe brain trauma. Both markers appeared significantly reduced in the TBI group suffering from severe trauma, which is in agreement with a significant downregulation of miR-16 we had observed in the cerebellum of deceased persons after fatal TBI.

Recently, authors pointed to limitations in the quality of RNA isolated from postmortem human brain tissue [40]. Mean RIN values of 2.8 (ranging from 1.0 to 6.2) indicated that a severe RNA degradation occurred. In contrast, our RIN values ranged from 6.0 to 9.1 (mean value 7.4), suggesting that postmortem in vivo RNA degradation (up to 6 days after death) might be a minor issue, in particular, when applying modern molecular biological methodologies such as whole-genome microarrays and qRT-PCR. A nearly 3-fold higher postmortem interval (PMI) did not influence the RIN quality (PMI median, 58 vs. 22 h cited by Koppelkamm and colleagues [40]).

We were curious about the impact of age at biosampling, the survival time, and the differences in the postmortem interval among our groups. We examined their impact in order to discriminate between both groups. Fortunately, all three confounders did not have any significant influence, which is in line with the low number of differentially expressed genes found among both groups, indicating close similarities in transcriptional control irrespective of, e.g., age differences.

Our study has some limitations. In particular, the number of examined individuals is low. Therefore, the promising concordance, PPV and NPV, should be taken with caution and definitely have to be validated in another and independent group. We were curious about the large inter-individual variance of gene expression data and the impact of environmental factors not covered by our study design and interfering with our results. However, the very consistent normalized Ct values and low standard deviations measured among all group members and the low number of differentially expressed genes argues against a strong impact of these factors on our results. The high consistency of gene expression measurements performed by whole-genome microarrays and qRT-PCR lessens concerns that our findings are due to methodological artifacts.

Taken together, our results reveal that cerebral hypoxia after a frontal cortex injury causes gene expression changes that can be measured in the cerebellum. The understanding of these gene regulation mechanisms expands our knowledge about hypoxia after TBI. Our novel miRNA markers are useful for postmortem studies where the tissue can be degraded and other markers failed.

In further studies, it should be analyzed whether the concentration of our novel hypoxia markers vary in dependence of the severity of a brain injury. In this case, the markers could be used to estimate the severity of a brain injury. Furthermore, the novel hypoxia markers could be new targets for future therapies.

## Electronic supplementary material

Below is the link to the electronic supplementary material.

Online Resource 1 (DOCX 36 kb)
