# Supplementary material for: Detection of hypoxia markers in the cerebellum after a traumatic frontal cortex injury: a human postmortem gene expression analysis
Source: Int J Legal Med. 2014 Nov 29;129(4):701–7. doi: 10.1007/s00414-014-1129-3 (PMC4475240; doi:10.1007/s00414-014-1129-3)
Supplement: Supplementary file 1 — (DOCX 36 kb) [file 414_2014_1129_MOESM1_ESM.docx]

Detection of Hypoxia Markers in the Cerebellum after a Traumatic Frontal Cortex Injury: a Human Postmortem Gene Expression Analysis

International Journal of Legal Medicine

Schober K., Ondruschka B., Dreßler J. and Abend M.

E-mail corresponding Author: kristinschober@bundeswehr.org

| SUPPLEMENTAL TABLE 1. MRNA AND MICRORNA DATA | | | | | | | | | |
| --- | --- | --- | --- | --- | --- | --- | --- | --- | --- |
|  | **TBI** | **control** | **cut off** |  |  |  |  |  |  |
| **variable** | **n** | **n** | **value** | **p-value** | **sens.** | **spec.** | **PPV** | **NPV** | **conc.** |
| *transcriptional changes* | | |  |  |  |  |  |  |  |
| HSPA12B | 8 | 7 | 8.30 | 0.002 | 100.0 | 85.7 | 88.9 | 100.0 | 96.4 |
| HSD11B1 | 8 | 7 | 5.70 | 0.006 | 57.1 | 100.0 | 72.7 | 100.0 | 89.3 |
| FOSB | 8 | 7 | 12.60 | 0.026 | 42.9 | 100.0 | 100.0 | 66.7 | 85.7 |
| IL6 | 8 | 7 | 12.50 | 0.058 | 85.7 | 75.0 | 75.0 | 85.7 | 76.8 |
| GRIA3 | 8 | 7 | 4.70 | 0.07 | 100.0 | 57.1 | 72.7 | 100.0 | 75.0 |
| S100B | 8 | 7 | 2.40 | 0.16 | 87.5 | 71.4 | 77.8 | 83.3 | 73.2 |
| RGS6 | 8 | 7 | 7.40 | 0.19 | 75.0 | 71.4 | 75.0 | 71.4 | 69.6 |
| NTRK2 | 8 | 7 | 3.30 | 0.28 | 100.0 | 42.9 | 66.7 | 100.0 | 64.3 |
| BCAT | 8 | 7 | 7.80 | 0.29 | 75.0 | 85.7 | 85.7 | 75.0 | 71.4 |
| CASP3 | 8 | 7 | 5.30 | 0.30 | 100.0 | 42.9 | 66.7 | 100.0 | 64.3 |
| GFAP | 8 | 7 | -0.40 | 0.39 | 75.0 | 42.9 | 60.0 | 60.0 | 60.7 |
| PRPH | 8 | 7 | 14.50 | 0.40 | 71.4 | 75.0 | 71.4 | 75.0 | 62.5 |
| GADD45B | 8 | 7 | 5.10 | 0.42 | 71.4 | 62.5 | 62.5 | 71.4 | 66.1 |
| ARPC5 | 8 | 7 | 5.75 | 0.68 | 71.4 | 62.5 | 71.4 | 62.5 | 66.1 |
| *post-transcriptional changes* | | |  |  |  |  |  |  |  |
| miR-138 | 8 | 7 | 11.95 | 0.007 | 71.4 | 100.0 | 100.0 | 80.0 | 89.3 |
| miR-504 | 7 | 6 | 16.00 | 0.008 | 83.3 | 85.7 | 83.3 | 85.7 | 90.5 |
| miR-16 | 8 | 4 | 17.13 | 0.010 | 100.0 | 75.0 | 88.9 | 100.0 | 90.6 |
| miR-376a | 8 | 7 | 15.19 | 0.016 | 100.0 | 62.5 | 70.0 | 100.0 | 83.9 |
| miR-195 | 8 | 7 | 11.41 | 0.018 | 71.4 | 100.0 | 100.0 | 80.0 | 87.5 |
| miR-370 | 7 | 7 | 16.50 | 0.020 | 85.7 | 100.0 | 100.0 | 88.9 | 85.7 |
| miR-320B | 7 | 6 | 16.10 | 0.022 | 100.0 | 57.1 | 66.7 | 100.0 | 85.7 |
| miR-135b | 8 | 7 | 12.03 | 0.026 | 71.4 | 87.5 | 83.3 | 77.8 | 83.9 |
| miR-744 | 8 | 7 | 15.42 | 0.027 | 85.7 | 75.0 | 75.0 | 85.7 | 85.7 |
| miR-324-5p | 8 | 7 | 16.60 | 0.028 | 71.4 | 87.5 | 83.3 | 77.8 | 82.1 |
| miR-455-3p | 8 | 7 | 17.40 | 0.034 | 57.1 | 100.0 | 100.0 | 72.7 | 82.1 |
| let-7a | 8 | 7 | 14.65 | 0.035 | 87.5 | 71.4 | 77.8 | 83.3 | 80.4 |
| miR-193b | 8 | 7 | 15.95 | 0.050 | 85.7 | 87.5 | 85.7 | 87.5 | 82.1 |
| TBI: traumatic brain injury; sens: sensitivity; spec: specificity; PPV: positive predictive value; NPV: negative predictive value; conc: concordance; formula for estimation: sensitivity = true positive / true positive + false negative; specificity = true negative / true negative + false positive; PPV = true positive / true positive + false positive; NPV = true negative / true negative + false negative and for cut off estimation: x = 0.5 (sensitivity + specificity). The cut off value is found at the maximal x-value (Linnet & Brandt (1986) Clin Chem 32: 1341−1346). | | | | | | | | | |

| SUPPLEMENTAL TABLE 2. TRANSCRIPTIONAL AND POST-TRANSCRIPTIONAL GENE CANDIDATES | | | | |  | |
| --- | --- | --- | --- | --- | --- | --- |
|  |  |  | *transcriptional gene candidates* |  | |  |
| **gene ID** | **gene name** | **chromo-some** | **gene function** | **published articles related to traumatic brain injury (TBI)** | | **literature reference** |
|  |  |  | ***G protein signaling*** |  | |  |
| RGS6 | regulator of G-protein signaling 6 | 14q24.2 | The RGS proteins inhibit G-protein signaling via activating the intrinsic GTPase activity of the G-protein alpha subunit. RGS6 interacts with SCG10 and promotes neuronal differentiation. RGS6 is detected in the granule cell layer of the cerebellum. RGS6 was identified as a key regulator of gamma-aminobutyric acid (B) receptors (GABA(B)R) signaling in cerebellum. RGS6 knockout mice showed an abnormal gait and ataxia improved by treatment with a GABA(B)R antagonist. | no cited article | | De Vries et al. (1995) Proc Natl Acad Sci U S A 92: 11916−11920  Liu et al. (2002) J Biol Chem 277: 37832−37839  Maity et al. (2012) J Biol Chem 287: 4972−4981 |
|  |  |  | ***transcription factor*** |  | |  |
| FOSB | FBJ murine osteosarcoma viral oncogene homolog B | 19q13.32 | FOSB encodes a leucine zipper protein and dimerizes with JUN proteins to the AP-1 complex. The members of the FOS family are c-fos, FOSB, FOSB2, deltaFOSB2, Fra-1 and Fra-2. FosB/JunD AP-1 transcription factor complex is involved in glutamate-mediated excitotoxicity. | No cited article was found to FOSB. The following articles are related to c-fos: After TBI c-fos expression was higher in cortex than c-fos expression after TBI combined with hypotension. In the frontal cortex, cerebellum and hippocampus, an increase was shown in c-fos mRNA levels following TBI. Hypoxia potentiates the c-fos mRNA in the cerebellum. There was no induction of the c-Fos protein in the cerebellum after ≤ 3 h following TBI. Immediate early genes (like c-fos and zif/268) are markers of TBI in the cerebral cortex and could play a role in neurodegeneration and/or glial activation. The c-fos marker revealed a specific severity- and age-dependent pattern in hippocampus following TBI. c-Fos was upregulated in pericontusional tissue from TBI patients. Patients with poorer outcomes had a higher expression of c-Fos and c-Jun. Activation of NMDA receptors induced c-fos. Higher NMDA concentrations produced necrosis and lower concentrations induced apoptosis. | | Patel et al. (1990) Nature 347: 572−575  Lidwell et al. (2000) J Neurosci Res 62: 427−439  Awasthi et al. (2003) Neurosci Lett 345: 29−32  Dave et al. (1997) Neuroreport 8: 395−398  Fukuda et al. (1996) J Neurotrauma 13: 255−266  Dutcher et al. (1999) Neurol Res 21: 234−242  Giza et al. (2002) J Neurotrauma 19: 387−402  Michael et al. (2005) J Clin Neurosci 12: 284−290  Whitfield et al. (2000) Neurol Res 22: 138−144  Hasegawa et al. (1998) Brain Res 785: 262−278 |
|  |  |  | ***neuronal signal transduction*** |  | |  |
| GRIA3 | glutamate receptor, ionotropic, AMPA 3 (synonym: GluA3) | Xq25 | Binding of the L-glutamate to GRIA3 induces the opening of the cation channel, and thereby converts the chemical signal to an electrical impulse. GluA3-deficient mice showed an increase in isolation-induced male aggression, in sociability, social interactions and minor deficits in motor and balance function. | no cited article | | Hollmann et al. (1991) Science 252: 851−853  Adamczyk et al. (2012) Behav Brain Res 229: 265−272 |
|  |  |  | ***cell cycle control*** |  | |  |
| GADD45B | growth arrest and DNA-damage-inducible, beta (synonym: MyD118) | 19p13.3 | MyD118 regulates growth arrest and apoptosis. GADD45B interacts with Cdk1 and cyclinB1, resulting in inhibition of the Cdk1/cyclinB1 complex. GADD45b cooperates in activation of S and G2/M checkpoints following genotoxic stress. GADD45b enhances the interaction between p38 and retinoblastoma protein phosphorylation during Fas-induced apoptosis. GADD45b is increased in the brains of psychotic patients maybe due to its inability to access gene promoter regions. | no cited article | | Selvakumaran et al. (1994) Mol Cell Biol 14: 2352−2360  Vairapandi et al. (2002) J Cell Physiol 192: 327−338  Cho et al. (2010) J Biol Chem 285: 25500−25505  Gavin et al. (2012) Neuropsychopharmacology 37: 531−542 |
| S100B | S100 calcium binding protein B | 21q22.3 | The highest levels of S100B are found in the brain, mainly in astrocytes. The protein is implicated in cell cycle control and promotes viability, neurite extension and morphological differentiation. S100B stimulates the proliferation of astroglia in nanomolar concentrations. Higher S100B concentrations cause apoptosis via stimulation of reactive oxygen species production and inhibition of the pro-survival kinase and extracellular signal-regulated mitogen-activated protein kinase (ERK)1/2. | S100B in cerebrospinal fluid (CSF) and serum were significantly higher in TBI vs. controls. An increased CSF S100B level (>2000 ng/ml) in the early postmortem period might be a sign of fatally severe brain damage. The serum level was increased for acute deaths from head injury. S100B gene expression was significantly higher at the cerebellar trauma site vs. cerebral trauma. | | Kahn et al. (1983) Am J Clin Pathol 79: 341−347  Kato et al. (1985) Biochem Biophys Acta 842: 146−150  Kligman et al. (1985) Proc Natl Acad Sci U S A 82: 7136−7139  Kligman et al. (1987) Brain Res 430: 296-300  Van Eldik et al. (1988) J Biol Chem 263: 7830−7837  Winningham-Major et al. (1989) J Cell Biol 109: 3063−3071  Selinfreund et al. (1991) Proc Natl Acad Sc. U S A 88: 3554−3558  Sorci et al. (2004) J Cell Physiol 199: 274−283  Ondruschka et al. (2013) J Neurotrauma 30: 1862−1871  Li et al. (2009) Leg Med (Tokyo) 11 Suppl 1: S273−S275  Li et al. (2006) Leg Med (Tokyo) 8: 71−77  Staffa et al. (2012) J Neurotrauma 29: 2716−2721 |
|  |  |  | ***inflammation*** |  | |  |
| IL6 | interleukin 6 (interferon, beta 2), (synonym: BSF-2) | 7p15.3 | Human B-cell differentiation factor (BSF-2) induces the final maturation of B cells into immunoglobulin-secreting cells. IL-6 stimulates various acute-phase proteins including C-reactive protein, β2-fibrinogen, amyloid protein, haptoglobin, hemopexin. | The changes of serum IL-6 levels showed positive correlation to the severity of TBI patients. The serum IL-6 reached the maximum level on the 7th day in TBI patients with a GCS score of 9-12. The severity of trauma correlated with the expression and activation of p38 mitogen-activated protein kinase, as well as the elevation of IL-6 expression. Anesthesia during the induction of experimental brain trauma did not influence IL-6 expression. | | Hirano et al. (1986) Nature 324: 73−76  Andus et al. (1987) FEBS Lett 221: 18−22  Gauldie et al. (1987) Proc Natl Acad Sci U S A 84: 7251−7255  He et al. (2009) Nan Fang Yi Ke Da Xue Xue Bao 29: 999−1001  Wang et al. (2010) J Surg Res 161: 119−125  Luh at al. (2011) PLoS One 6: e19948 |
|  |  |  | ***apoptosis*** |  | |  |
| CASP3 | caspase 3, apoptosis-related cysteine peptidase | 4q34 | CASP3 is a member of the cysteine-aspartic acid protease (caspase) family and implicated in the apoptosis pathway. | Caspase 3 mRNA expression was increased in the injured cortex and hippocampus and about twice higher than in the control group. After TBI, caspase inhibition suppressed elevations in amyloid-beta peptide. For acute death, the expression of CASP3 in the cerebellar trauma site was increased compared to the controls or the cerebral trauma site. | | Alnemri et al. (1996) Cell 87: 171  Fernandes-Alnemri et al. (1994) J Biol Chem 269: 30761−30764  Yang et al. (2002) Chin J Traumatol 5: 250−253  Abrahamson et al. (2006) Exp Neurol 197: 437−450  Staffa et al. (2012) J Neurotrauma 29: 2716−2721 |
| HSPA12B | heat shock 70kD protein 12B | 20p13 | The protein is an atypical heat Hsp70 protein and a distant member of the mammalian Hsp70 family. HSPA12B is predominantly expressed in endothelial cells and required for angiogenesis. HSP12B expression is found in atherosclerotic lesions. Transgenic mice overexpressing human HSPA12B show significant decreases in cell injury and apoptosis after ischemia/reperfusion injury. HSPA12B protects the brains through activation phosphatidylinositol 3 kinase and serine/threonine proteinkinase Akt signaling and suppressing c-jun N-terminal kinase and p38 activation. A downregulation of HSPA12B inhibits the expression of active caspase-3 and prevents apoptosis. | no cited article | | Steagall et al. (2006) Arterioscler Thromb Vasc Biol 26: 2012−2018  Han et al. (2003) Proc Natl Acad Sci U S A 100: 1256−1261  Ma et al. (2013) Biochem Biophys Acta 1832: 57−66  Kang et al. (2013) Neurochem Res 38: 311−320 |
|  |  |  | ***steroid hormone biosynthesis*** |  | |  |
| HSD11B1 | hydroxy-steroid (11-beta) dehydro-genase 1 | 1q32.2 | The enzyme that is highly expressed in the liver, adipose and central nervous system promotes the conversion of glucocorticoids corticosterone and cortisol to inactive 11 keto-products (11-dehydrocorticosterone, cortisone). The enzyme can also catalyze a reverse reaction. In neuronal cells HSD11B1 acts as reductase and dehydrogenase. A HSD11B1 deficiency leads to improvements of cognitive functions. | The drugs metyrapone and carbenoxolone showed inhibitory effects on HSD11B1 in hippocampus after TBI in rats, resulting in inhibition of glucocorticoid targets like neuropeptide Y and tyrosine hydroxylase. | | Agarwal et al. (1989) J Biol Chem 264: 18939−18943  Albertin et al. (2002) Int J Mol Med 9: 495−498  Lakshmi et al. (1988) Endocrinology 123: 2390−2398  Lakshmi et al. (1991) Endocrinology 128: 1741−1748  Moisan et al. (1990) Endocrinology 127: 1450−1455  Ricketts et al. (1998) J Clin Endocrinol Metab 83: 1325−1335  Tannin et al. (1991) J Biol Chem 266: 16653−16658  Jellinck et al. (1999) J Steroid Biochem Mol Biol 71: 139−144  Yau et al. (2001) Proc Natl Acad Sci U S A 98: 4716−4721  Hellmich et al. (2013) PLoS One 8: e53230 |
|  |  |  | ***cell growth*** |  | |  |
| ARPC5 | actin related protein 2/3 complex, subunit 5, 16kDa | 1q25.3 | The Arp2/3 protein complex controls the actin polymerization in cells. ARPC5 is related with neurite outgrowth. | no cited article | | Millard et al. (2003) Cell Motil Cytoskeleton 54: 81−90  Inagaki et al. (2001) Nat Neurosci 4: 781−782 |
| BCAT1 | branched chain amino-acid transaminase 1, cytosolic | 12p12.1 | This enzyme catalyzes the transamination of the essential branched chain amino acids (leucine, isoleucine and valine) with alpha-ketoglutarate that is essential for cell growth. Overexpression of BCAT1 leads under serum deprivation to cell death. BCAT1 is restricted to neurons. The BCAT1-neurons are either GABA-ergic or glutamatergic and involved in glutamate production and glutamate release during excitation. | no cited article | | Eden et al. (1996) J Biol Chem 271: 20242−20245  Naylor et al. (1980) Cell Genet 6: 641−652  Eden et al. (1999) FEBS Lett 457: 255−261  Hull et al. (2012) J Neurochem 123: 997−1009 |
| NTRK2 | neurotrophic tyrosine kinase, receptor, type 2 (synonym: TrkB) | 9q22.1 | TrkB and his ligand brain-derived neurotrophic factor (BDNF) have been implicated in regulating neural axon growth as well as in neuroprotection, neuronal repair and recovery after traumatic brain injury. | The mRNA expression of BDNF and TrkB decreased in the hippocampus in the border zone to the injury while there was an increase in mRNA expression at the contralateral site. The mRNA TrkB level was increased in the dentate gyrus following a lateral fluid-percussion brain injury. In the injured cortex and adjacent cortex, the expression of TrkB mRNA was significantly decreased. The decrease was seen in cortical regions that contain degenerating neurons. For acute death, the expression of TrkB in the cerebellar trauma site was increased compared to the controls or the cerebral trauma site. | | Fayard et al. (2005) J Neurosci Res 80: 18−28  Hicks et al. (1998) Brain Res Mol Brain Res 59: 264−268  Hicks et al. (1999) J Neurotrauma 16: 501−510  Rostami et al. (2014) Brain Res 1542: 195−205  Staffa et al. (2012) J Neurotrauma 29: 2716−2721 |
|  |  |  | ***intermediate filaments*** |  | |  |
| PRPH | Peripherin | 12q13.12 | Peripherin is a member of the intermediate filament protein family. Peripherin plays a role in axon genesis following nerve injury. Focal ischemia induces peripherin accumulations in neurons of the cortex, thalamus and hippocampus. Estrogen enhances nerve growth factor-stimulated neurite sprouting as well as peripherin expression. | no citied article | | Portier et al. (1983) Dev Neurosci 6: 335−344  Oblinger et al. (1989) J Neurosci 9: 3766−3775  Troy et al. (1990) Brain Res 529: 232−238  Beaulieu et al. (2002) Brain Res 946: 153−161  Gollapudi et al. (2001) Exp Neurol 171: 308−316 |
| GFAP | glial fibrillary acidic protein | 17q21 | GFAP is an intermediate filament protein that is expressed in the central nervous system including astrocytes. The protein is involved in glia scarring. | After penetrating ballistic brain injury activated astrocytes (GFAP-positive cells) were detected followed by microglial reactivity. GFAP positive cells increased significantly 12 hours after contusion, reached the maximum at the 4th day. Injured brains showed an increased number of GFAP-positive astrocytes and more intense GFAP reaction in comparison to control brains. GFAP did not show an increase in gene expression in the cerebral trauma site, contralateral site, hippocampus and cerebellar trauma site. | | Jacque et al. (1978) J Neurol Sci 35: 147−155  Smith et al. (1987) J Neurosci Res 18: 203−208  Williams et al. (2007) J Neuroinflammation 4: 17  Tao et al. (2000) Fa Yi Xue Za Zhi 16: 137−138  Regner et al. (2001) J Neurotrauma 18: 783−792  Staffa et al. (2012) J Neurotrauma 29: 2716−2721 |
|  |  |  | *post-transcriptional gene candidates* |  | |  |
|  | **miRNA ID** | **chromo-some** | **miRNA function** | **published articles related to TBI** | |  |
|  | miR-138 | miR138-1: 3p21.32, miR138-2: 16q13 | 560 mRNA targets are found for miR-138 (www.Targetscan.org). (www.Targetscan.org). miR-138 acts as a tumor suppressor of the oncogene vimentin. | miR-138 was reduced in cerebral cortex after TBI in rats. | | Yamasaki et al. (2012) Int J Oncol 41: 805−817  Lei et al. (2009) Brain Res 1284: 191−201 |
|  | miR-504 | Xq26.3 | 211 conserved targets are found for miR-504 (www.Targetscan.org). | no cited article | |  |
|  | miR-16 | 13q14 | 1273 conserved targets are found for miR-16. One Target of miR-16 is **GFAP** (www.Targetscan.org). miR-16 regulates cell proliferation, cell cycles progression and apoptosis. | miR-16 was used as a specific marker to distinguish a TBI from a polytrauma as well as a mild brain trauma from a severe brain trauma. In case of severe trauma, the marker miR-16 was significantly reduced and elevated in case of a mild trauma. | | Cimmino et al. (2005) Proc Natl Acad Sci U S A 102: 13944−13949  Kaddar et al. (2009) Biol Cell 101: 511−524  Linsley et al. (2007) Mol Cell Biol 27: 2240−2252  Redell et al. (2010) J Neurotrauma 27: 2147−2156 |
|  | miR-376a | 14q32.31 | 212 conserved targets are found for miR-376a. | no cited article | |  |
|  | miR-195 | 17p13.1 | 1273 conserved mRNA targets are found for miR-195 (www.Targetscan.org). BCL2 is one target of miR-195 (http://pictar.mdc-berlin.de). miR-195 decreases BCL2 protein levels and promotes apoptosis. An overexpression of miR-195 causes a downregulation of amyloid precursor protein (APP) and β-site APP cleaving enzyme 1 (BACE1) protein and results in reduced dementia vulnerability. miR-195 regulates the expression of BDNF and alters the expression of downstream GABAergic transcripts in schizophrenia. | no cited article | | Chen et al. (2011) Am J Nephrol 34: 549−559  Liu et al. (2010) Biochem Biophys Res Commun 400: 236−240  Singh et al. (2012) J Cell Sci 125: 1568−1578  Ai et al. (2013) J Neurosci 33: 3989−4001  Zhu et al. (2012) Brain Res Bull 88: 596−601  Guo et al. (2010) BMC Syst Biol 4: 10  Mellios et al. (2008) Hum Mol Genet 17: 3030−3042  Shi et al. (2012) J Psychiatr Res 46: 198−204 |
|  | miR-370 | 14q32.2 | 385 conserved targets are found for miR-370. Targets of miR-370 are **GADD45B and FOSB** (www.Targetscan.org). | no cited article | |  |
|  | miR-320B | 1 | 786 conserved targets are found for miR-320B (www.Targetscan.org). miR-320B is a possible regulator of human-specific neural development. | no cited article | | Somel et al. (2011) PLoS Biol 9: e1001214 |
|  | miR-135b | 1q32.1 | 718 conserved targets are found for miR-135b. | no cited article | |  |
|  | miR-744 | 17p12 | 100 mRNA targets are found for miR-744 (www.Targetscan.org). One target is JunB that dimerizes with FOSB to the AP-1 complex. | no cited article | | Patel et al. (1990) Nature 347: 572−575 |
|  | miR-324-5p | 17p13.1 | 142 mRNA targets are found for miR-324-5p (http://pictar.mdc-berlin.de/). | miR324-5p was decreased in cerebral cortex after TBI. | | Lei et al. (2009) Brain Res 1284: 191−201 |
|  | miR-455-3p | 9q32 | 199 conserved targets are found for miR-455-3p (www.Targetscan.org). | no cited article | |  |
|  | let-7a | 9q22.32 | Targets of let-7a are **CASP3 and RGS6** (www.Targetscan.org). | no cited article | |  |
|  | miR-193b | 16p13.12 | 221 conserved targets are found for miR-193b (www.Targetscan.org). | no cited article | |  |
